# Social network analysis of key stakeholders in Brucellosis prevention in Western Iran

**DOI:** 10.1186/s41182-025-00693-w

**Published:** 2025-02-05

**Authors:** Meysam Behzadifar, Mohammad Yarahmadi, Ahad Bakhtiari, Sahar Kargar, Saeed Shahabi, Samad Azari, Mariano Martini, Masoud Behzadifar

**Affiliations:** 1 Social Determinants of Health Research Center, Lorestan University of Medical Sciences, Khorramabad, Iran; 2 Department of Medical Parasitology and Mycology, Lorestan University of Medical Sciences, Khorramabad, Iran; 3 Health Equity Research Center (HERC), Tehran University of Medical Sciences (TUMS), Tehran, Iran; 4 Department of Health Management, Policy and Economics, School of Public Health, Tehran University of Medical Sciences (TUMS), Tehran, Iran; 5 Health Policy Research Center, Institute of Health, Shiraz University of Medical Sciences, Shiraz, Iran; 6 Hospital Management Research Center, Health Management Research Institute, University of Medical Sciences, Tehran, Iran; 7 Department of Health Sciences, University of Genoa, Genoa, Italy

**Keywords:** Brucellosis, Social network analysis, Iran, One Health, Health policy

## Abstract

**Background:**

Brucellosis remains a persistent public health challenge in Iran, particularly in rural regions such as Lorestan province, due to systemic, economic, and cultural barriers. Effective disease control requires multisectoral collaboration among stakeholders. This study aimed to map the stakeholder network involved in brucellosis prevention in Lorestan province, identify gaps in coordination, and provide actionable recommendations for improving control strategies.

**Methods:**

This cross-sectional study employed social network analysis (SNA) to explore the relationships among key stakeholders in brucellosis prevention. Data were collected through a structured questionnaire administered to 75 experts from various sectors, including health, veterinary, agriculture, and non-governmental organizations. The SNA evaluated network density, clustering coefficient, and centrality metrics to determine the levels of collaboration and influence among stakeholders.

**Results:**

The analysis revealed a moderately dense network (density: 0.2745; clustering coefficient: 0.2839) with central roles played by the Veterinary Organization of Lorestan Province, Lorestan University of Medical Sciences, and the Ministry of Agriculture. These organizations exhibited high levels of influence, support, and interest in brucellosis prevention. However, limited involvement of community-based organizations and environmental agencies was identified, highlighting a critical gap in grassroots engagement. Fragmented coordination was particularly evident in rural areas, where traditional livestock practices, inadequate veterinary services, and the consumption of unpasteurized dairy products perpetuate disease transmission. Economic constraints, such as the high cost of vaccines, along with limited public awareness, further hinder effective control efforts.

**Conclusions:**

Brucellosis prevention in Lorestan province requires a comprehensive, multisectoral approach. The adoption of a One Health framework can improve collaboration among stakeholders, enhance resource allocation, and address systemic barriers. Community engagement and intersectoral coordination are essential for improving public awareness and compliance with preventive measures. These findings provide a foundation for developing a National Brucellosis Control Program and inform strategies to mitigate zoonotic diseases in similar high-risk regions.

**Supplementary Information:**

The online version contains supplementary material available at 10.1186/s41182-025-00693-w.

## Introduction

Brucellosis, a zoonotic disease of global significance, affects over 500,000 people annually, presenting substantial health, economic, and social challenges in both developed and developing countries [1]. The disease reduces livestock productivity**,** causing financial losses amounting to millions of dollars annually, particularly in regions heavily reliant on agriculture [2]. For affected individuals, brucellosis often manifests as a chronic condition characterized by debilitating symptoms such as fever, joint pain, and fatigue**,** resulting in prolonged disability and reduced quality of life [3]. These health challenges place significant pressure on healthcare systems**,** especially in resource-limited settings [4]. Moreover, the economic burden of diagnostic tests, prolonged treatments, and hospitalizations intensifies financial strain on families and governments [5]. At a global scale, brucellosis disrupts international trade, as restrictions on infected livestock and contaminated products lead to economic losses for agricultural and food sectors [6].

In Iran, brucellosis remains a major public health challenge, with incidence rates in some regions far exceeding the national average [7]. The western provinces—including Lorestan, Kermanshah, Kurdistan, Hamedan, and Ilam—are particularly vulnerable due to their heavy reliance on livestock farming and the widespread use of traditional agricultural practices [8]. Cultural factors, such as the consumption of unpasteurized dairy products, further increase the risk of transmission [9]. Additionally, systemic challenges—such as limited access to veterinary services, inadequate public health awareness, and gaps in disease surveillance and control programs—exacerbate the situation. For instance, suboptimal vaccination coverage and the lack of diagnostic services in remote areas hinder timely interventions [10].

The economic burden of brucellosis in Iran is substantial [11]. Combating the disease necessitates significant investments in healthcare services, disease surveillance, and vaccination programs, placing considerable strain on the country’s health system [12]. Despite these efforts, the persistence of the disease underscores critical gaps in prevention and control policies [13]. Bridging these gaps requires a comprehensive understanding of the socioeconomic impacts of brucellosis and the barriers faced by healthcare providers and policymakers in implementing effective interventions [14].

This study aims to address these critical gaps by analyzing the roles and interactions of key stakeholders involved in brucellosis prevention in Western Iran. By utilizing region-specific data, the research seeks to provide actionable insights for policymakers and health managers on optimizing resources, enhancing coordination, and improving the implementation of prevention programs. The findings are expected to contribute to reducing the burden of brucellosis and strengthening public health interventions in these high-risk regions.

## Methods

### Social network analysis (SNA)

SNA is a methodological approach used to study and map relationships and interactions among individuals, organizations, or entities within a network [15]. By focusing on the structure and dynamics of these connections, SNA provides valuable insights into influence, power, and the flow of information within a system [16]. In health systems, SNA has been widely applied to explore collaborative dynamics, identify influential stakeholders, and optimize resource allocation [17]. For instance, it has been utilized to understand infectious disease transmission, enhance health program implementation, and improve policy coordination among diverse actors [18].

SNA was selected for this study due to its ability to address the complex, multifaceted nature of brucellosis prevention, which requires coordinated efforts from government agencies, healthcare providers, veterinarians, and communities [19]. Brucellosis prevention involves not only biological and medical interventions but also the integration of social, cultural, and policy dimensions [20]. By mapping and analyzing social networks in Western Iran, SNA provides a deeper understanding of stakeholder roles, influences, and interactions, offering actionable insights to strengthen partnerships, address systemic barriers, and enhance prevention efforts [21].

### Data collection

#### Study timeline

The study was conducted from January 2024 to August 2024 in two phases. The first phase focused on the identification of key stakeholders involved in brucellosis prevention. This was achieved through a comprehensive document review, which included official reports from organizations such as the Ministry of Health and Medical Education (MoHME) and the Veterinary Organization of Iran, as well as studies from international bodies like the World Health Organization (WHO) and the Food and Agriculture Organization (FAO). Databases such as PubMed, Scopus, Embase, ISI Web of Science, MagIran, and Scientific Information Database (SID) were searched using keywords like “brucellosis”, “prevention”, and “control”, covering the period from 2000 to 2024. An initial list of stakeholders—comprising government agencies, non-governmental organizations (NGOs), research centers, and local and international organizations—was refined through expert consultations with 10 professionals in health, veterinary medicine, and policy.

#### Participant selection

The study employed a purposive sampling approach to select participants based on predefined inclusion and exclusion criteria. Inclusion criteria required participants to be experts with at least 5 years of experience in brucellosis prevention or related fields, ensuring they possessed sufficient depth of knowledge and practical expertise. Participants were also required to be representatives from key sectors involved in brucellosis prevention, including health (e.g., public health officials, epidemiologists), veterinary medicine (e.g., veterinarians, animal health specialists), agriculture (e.g., livestock managers, agricultural extension officers), and non-governmental organizations (NGOs) actively engaged in zoonotic disease control programs. Exclusion criteria were applied to individuals with less than 5 years of experience in the field, as they were deemed less likely to provide the level of insight required for the study. Additionally, representatives from organizations not directly involved in brucellosis prevention, such as those focused solely on unrelated health or environmental issues, were excluded to maintain the relevance and focus of the study. These criteria ensured that the selected participants were well-qualified and representative of the stakeholder network involved in brucellosis prevention in Lorestan province.

#### Sampling method

A purposive sampling approach was employed to select 75 experts. Between one and three experts per organization were chosen to ensure diversity and prevent overrepresentation of any single entity. Participants were selected based on their expertise, organizational roles, and involvement in brucellosis prevention initiatives.

#### Sample size calculation

The sample size was determined based on the principle of saturation, where data collection continues until no new themes or insights emerge. Given the complexity of the stakeholder network and the need for diverse perspectives, a sample size of 75 was deemed adequate to capture the breadth of stakeholder interactions and roles. This sample size was also calculated using the Cochrane formula for sample size estimation in population studies:

$$\text{n}= \frac{{\text{Z}}^{2}\times \text{p }(1-\text{p})}{{\text{e}}^{2}} = \frac{{(1.96)}^{2}\times 0.5 (1-0.5)}{(0.113{)}^{2}} = 75.$$

This calculation ensures that the sample size is statistically robust and aligns with similar SNA studies in public health.

#### Questionnaire development and validation

A questionnaire was developed, listing stakeholders and incorporating four evaluation items: influence, interest, power, and level of support, each scored from 0 to 100. The questionnaire was reviewed by seven experts for clarity, relevance, and comprehensiveness, and its reliability was tested using the test–retest method, yielding a Cronbach’s alpha of 0.85, which ensured internal consistency. The finalized questionnaire was distributed via secure links, and participants scored stakeholders while also suggesting additional relevant actors. Data were cross-validated against document review findings to ensure accuracy and completeness.

### Data analysis

The data were analyzed using R software (Version 4.4.1). Key network metrics provided insights into stakeholder roles, interactions, and the flow of resources. Centrality metrics included degree centrality, which measures the number of direct ties and indicates coordination roles; closeness centrality, which reflects proximity to others and enables faster communication; betweenness centrality, which highlights bridging roles and influence over information flow; eigenvector centrality, which assesses influence within the network, weighted by connection quality; hub metric, which identifies central actors directing flows within the network; and PageRank, which measures importance based on incoming links, highlighting decision-making power. Structural metrics included nodes, representing the total stakeholders; edges, representing relationships between stakeholders; network density, which reflects the proportion of actual versus possible connections and indicates collaboration efficiency; clustering coefficient, which measures the likelihood of sub-group formation and indicates trust and cooperation; average degree, which represents the mean direct connections per actor; triangles, which suggest the presence of strong collaborations; diameter, which measures the longest shortest path, representing the maximum number of steps required to connect any two nodes in the network and indicating communication efficiency; and average path length, which represents the mean shortest path steps between nodes.

#### Scoring and aggregation process

Scores for influence, interest, power, and support were aggregated for each stakeholder by calculating the mean score across participants. Discrepancies or outliers in scoring were addressed by reviewing individual responses and consulting with domain experts to ensure consistency. Composite scores in Fig. 5 were calculated using equal weighting for all four dimensions (influence, interest, power, and support) to ensure a balanced assessment of stakeholder roles.

To ensure the robustness of our findings, we conducted statistical validation and sensitivity analysis as part of the SNA.

### Statistical validation

We compared the observed stakeholder network with 1000 randomized networks generated using the Erdős–Rényi model, which assumes random connections between nodes [22]. This allowed us to assess the statistical significance of key network metrics, such as density, clustering coefficient, and centrality measures. By comparing the observed metrics with those of the randomized networks, we determined whether the observed patterns of collaboration were statistically significant or could have occurred by chance.

### Sensitivity analysis

To evaluate the stability of the network properties, we performed sensitivity analysis by systematically removing key stakeholders and recalculating the network metrics. This approach allowed us to assess the impact of individual stakeholders on the overall network structure and identify critical actors whose removal significantly altered network properties. Additionally, we tested the robustness of the network under variations in edge weights to ensure that the findings were not overly sensitive to specific assumptions about stakeholder interactions.

## Results

A total of 58 participants completed the questionnaire, resulting in a response rate of 77.34%. The participants had a mean age of 43 ± 18 years and an average work experience of 16 ± 41 years. Of the participants, 41 (70.69%) were male, and 17 (29.31%) were female. The participants in this study identified 18 organizations as the key actors in brucellosis prevention programs in Lorestan province. These organizations include the Lorestan University of Medical Sciences (LUMS), Veterinary Organization of Lorestan Province (VOLS), Ministry of Agriculture, Jahad (AJ), Environmental Protection Organization (EPO), Lorestan Broadcasting Organization (LBO), Red Crescent Society (RCS), Non-Governmental Organizations (NGOs), Medical Society Basij (MSB), Governorship and District Administrations (GDA), Barakat Foundation (BF), Imam Khomeini Relief Committee (IKRC), Provincial Management and Planning Organization (PMPO), Universities and Research Centers (URC), Ministry of Cooperatives, Labor, and Social Welfare (CLSW), Welfare Organization (WO), Ministry of Education (OoE), Police (Po), and Guilds and Unions related to Animal Husbandry, Agriculture, and Food (GUAF). Local farmer associations and cooperatives, which are a sub-group of the Agricultural Jihad (AJ), also participated in the brucellosis prevention program. These groups provide services such as purchasing dairy or meat from livestock farmers, coordinating livestock vaccination campaigns, and collaborating with government agencies. Table 1 presents the names of these actors along with their activities related to the brucellosis program.

1. Power

**Table 1:** Key actors and their activities in brucellosis prevention in Lorestan province

No.	Actor	Organization type	Activities
1	Lorestan University of Medical Sciences (LUMS)	Governmental organizations	Responsible for prevention, diagnosis, and treatment programs for brucellosis; public education, vaccination programs, medical services for patients, and overseeing suspected case identification and control
2	Veterinary Organization of Lorestan Province (VOLS)	Governmental organizations	Monitors livestock health and vaccination, prevents disease transmission from animals to humans, identifies infected livestock, and implements quarantine programs
3	Agriculture, Jahad (AJ)	Governmental organizations	Promotes hygienic principles in livestock farming, supports farmers, improves sanitary conditions, and provides strategies to reduce brucellosis prevalence among livestock
4	Environmental Protection Organization of Lorestan Province (EPO)	Governmental organizations	Monitors wild animal health, prevents outbreaks among wildlife, maintains ecosystem balance, and prevents disease transmission from wildlife to livestock and humans
5	Lorestan Broadcasting Organization (LBO)	Governmental organizations	Produces and broadcasts educational programs to raise public awareness about brucellosis, particularly in rural and deprived areas
6	Red Crescent Society (RCS)	Non-profit organizations	Conducts health education, screening, and medical examination programs in rural areas; raises public awareness through brochures and informative initiatives
7	Non-Governmental Organizations (NGOs)	Non-governmental organizations	Focuses on awareness and public education, informs about prevention, promotes health behaviors among farmers, and acts as intermediaries to communicate public needs to government bodies
8	Medical Society Basij (MSB)	Governmental organizations	Organizes jihadist camps, provides health and educational services in rural areas, and assists in diagnosing and identifying suspected cases of brucellosis
9	Governorship and District Administrations (GDA)	Governmental organizations	Acts as coordinating entities, provides resources and facilities for joint programs, and oversees disease prevention and control projects at provincial and district levels
10	Barakat Foundation (BF)	Non-profit organizations	Enhances awareness through economic empowerment projects, supports educational programs for farmers, and contributes to disease prevention by promoting livestock vaccination
11	Imam Khomeini Relief Committee (IKRC)	Non-profit organizations	Meets health and treatment needs of vulnerable individuals, assists low-income farmers with vaccination and hygiene, and educates supported families
12	Provincial Management and Planning Organization (PMPO)	Governmental organizations	Allocates budgets for control and research programs related to brucellosis, facilitating preventive program implementation
13	Universities and Research Centers (URC)	Research institutions	Engages in epidemiological studies, provides innovative solutions, and contributes to improving prevention and treatment strategies for brucellosis
14	Ministry of Cooperatives, Labor, and Social Welfare (CLSW)	Governmental organizations	Provides support to disabled brucellosis patients, including insurance and supportive services to reduce their economic burden
15	Welfare Organization (WO)	Governmental organizations	Offers rehabilitation and support services to patients with disabilities caused by brucellosis, improving their quality of life
16	Ministry of Education (OoE)	Governmental organizations	Educates students and families on brucellosis prevention methods through awareness programs in schools, reducing disease prevalence in communities
17	Police (Po)	Governmental organizations	Prevents livestock smuggling, controls borders, and stops the entry of infected animals into Lorestan, helping to control disease outbreaks
18	Guilds and Unions related to Animal Husbandry, Agriculture, and Food (GUAF)	Local municipalities	Promotes hygienic principles during production, distribution, and consumption, preventing the spread of brucellosis and reducing disease transmission risks

VOLS is the most powerful stakeholder, scoring 100, signifying its central role in livestock health and vaccination programs. LUMS and AJ follow with scores of 95 and 90, respectively, highlighting their critical roles in medical and agricultural aspects of brucellosis prevention. GUAF scored 85, emphasizing their importance in ensuring hygienic practices. Other organizations such as RCS, NGOs, and EPO have moderate power (scores between 50 and 60), reflecting their supplementary but significant contributions.

2. Support

VOLS and LUMS again top the list with scores of 100 and 95, respectively, showcasing their strong commitment to prevention and control programs. AJ and EPO scored 90 and 85, respectively, indicating active participation in promoting hygiene and monitoring disease spread among wildlife. Stakeholders such as NGOs and BF scored lower [20], reflecting limited direct involvement or resource allocation for brucellosis prevention efforts.

3. Influence

VOLS, AJ, and LUMS lead with scores of 100, 95, and 90, indicating their pivotal roles in shaping and implementing brucellosis-related policies. EPO and URC scored 85 and 60, highlighting their influence through ecological monitoring and research contributions. Organizations like NGOs, BF, and WO scored lower (20–10), suggesting they play a supportive rather than decisive role in influencing brucellosis policies.

4. Interest

LUMS scored the highest (100), underlining its dedicated involvement in prevention, diagnosis, treatment, and public education. VOLS, AJ, and EPO followed closely with scores of 95, 90, and 85, respectively, showing their significant interest in controlling the disease. NGOs, BF, and WO scored lower (30–20), reflecting less focused involvement or competing priorities.

The scores for each stakeholder across the four items are provided in supplementary 1.

### SNA analysis

**Fig. 1:**
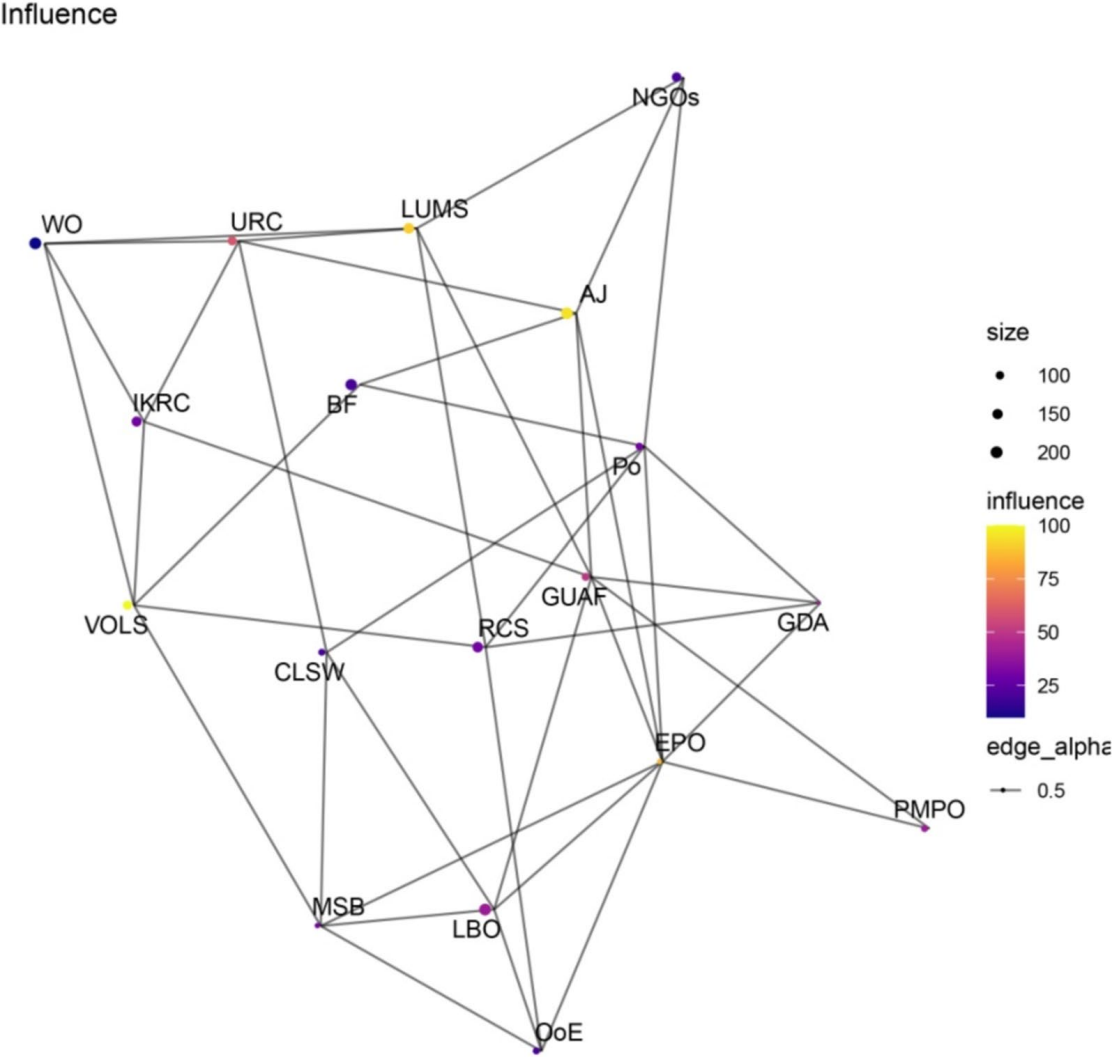
Network map illustrating the influence of stakeholders in brucellosis prevention

**Fig. 2:**
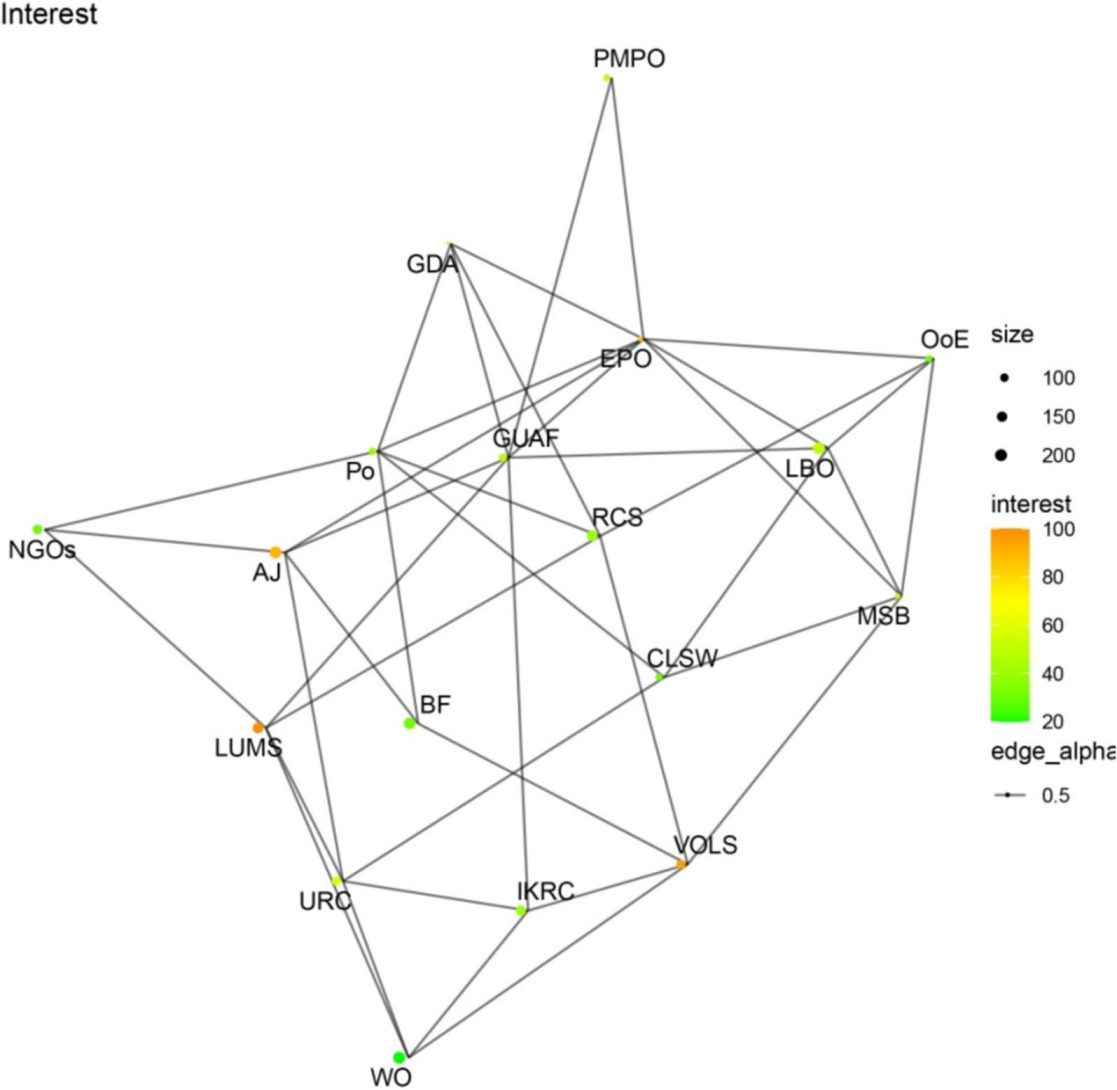
Network map depicting the interest of stakeholders in brucellosis prevention

**Fig. 3:**
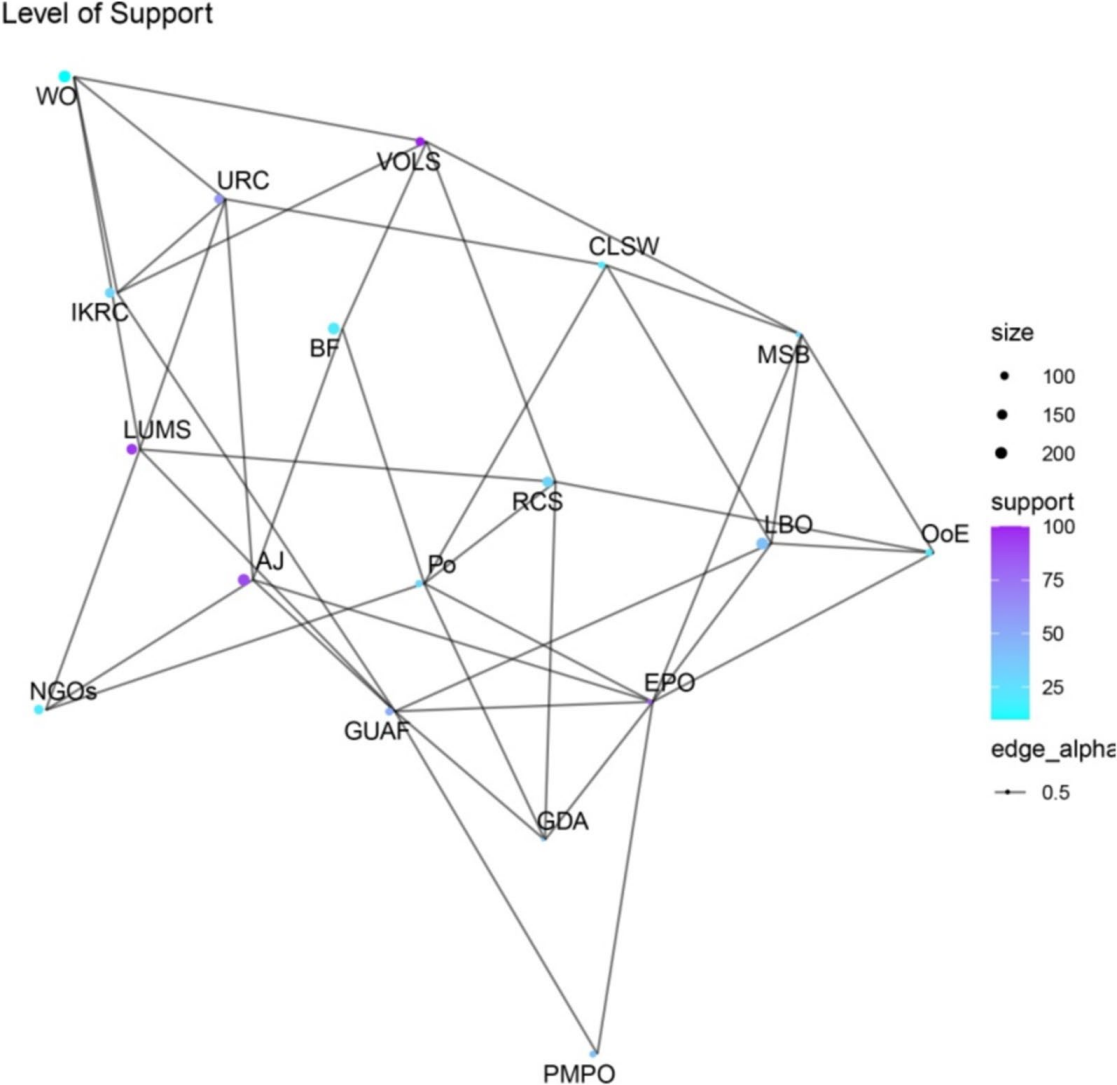
Network map showing the level of support from stakeholders in brucellosis prevention

**Fig. 4:**
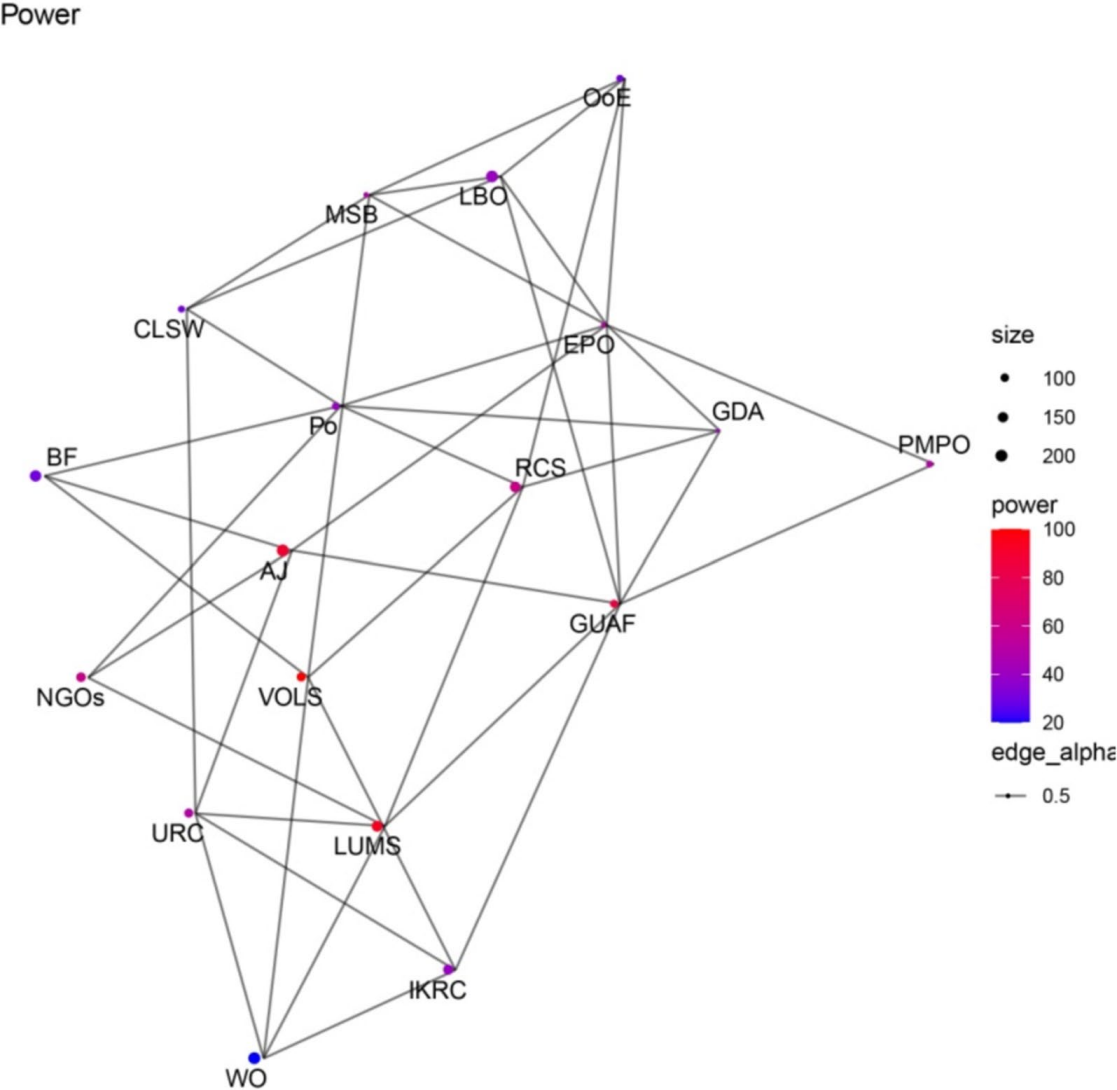
Network map representing the power of stakeholders in brucellosis prevention

Figures 1, 2, 3, 4 present network maps illustrating the influence, interest, level of support, and power of stakeholders involved in brucellosis prevention in Lorestan province. Table 2 presents the key network metrics for various stakeholders.

**Table 2:** Network metrics of key stakeholders in brucellosis prevention programs

Actor	Degree_Centrality	Closeness_Centrality	Betweenness_Centrality	Eigenvector_Centrality	Hub	PageRank
LUMS	0.2941	0.5484	0.0779	0.5228	0.5228	0.0596
VOLS	0.2941	0.5484	0.0848	0.4718	0.4718	0.06
AJ	0.2941	0.5667	0.0805	0.6021	0.6021	0.0596
EPO	0.4706	0.6296	0.1456	1	1	0.0898
LBO	0.2941	0.5312	0.0294	0.7094	0.7094	0.0579
RCS	0.2941	0.5484	0.0724	0.5681	0.5681	0.0588
NGOs	0.1765	0.4595	0.0136	0.3569	0.3569	0.0385
MSB	0.2941	0.5484	0.0501	0.6478	0.6478	0.0583
GDA	0.2353	0.5312	0.0157	0.6125	0.6125	0.0475
BF	0.1765	0.4857	0.0208	0.3468	0.3468	0.0386
IKRC	0.2353	0.5152	0.0331	0.4328	0.4328	0.0489
PMPO	0.1176	0.4359	0	0.3661	0.3661	0.0276
URC	0.2941	0.5312	0.0579	0.484	0.484	0.0597
CLSW	0.2353	0.5484	0.0457	0.4992	0.4992	0.0481
WO	0.2353	0.4722	0.015	0.3797	0.3797	0.0492
OoE	0.2353	0.4857	0.0169	0.5811	0.5811	0.0476
Po	0.3529	0.5667	0.1009	0.6721	0.6721	0.0701
GUAF	0.4118	0.6296	0.1469	0.8433	0.8433	0.0802

The social network analysis of stakeholders involved in the Brucellosis prevention program in Lorestan province highlights the roles and influence of key actors through various centrality metrics. Among the stakeholders, EPO and GUAF emerged as the most prominent, demonstrating their critical positions within the network. EPO exhibited the highest degree centrality (0.4706), indicating its extensive connections and pivotal role in fostering collaboration among stakeholders. Similarly, GUAF ranked second in degree centrality (0.4118), reflecting its significant connectivity. Both actors also recorded the highest closeness centrality scores (0.6296), signifying their ability to efficiently access and disseminate information throughout the network. In terms of betweenness centrality, GUAF (0.1469) and EPO (0.1456) held the highest values, underscoring their roles as key intermediaries facilitating communication and resource flow between other actors.

Eigenvector centrality further emphasized EPO as the most influential stakeholder (1.0000), reflecting its connections to other highly influential nodes, with GUAF (0.8433) closely following. Hub and authority scores confirmed EPO as the most critical hub, highlighting its capacity to connect with authoritative stakeholders. Similarly, the PageRank metric identified EPO (0.0898) and GUAF (0.0802) as the most significant actors, reinforcing their overall importance within the network. These findings indicate that EPO and GUAF play pivotal roles in the Brucellosis prevention program, serving as key facilitators of collaboration, information exchange, and decision-making. Engaging and supporting these stakeholders is essential for optimizing the program’s effectiveness and achieving its objectives. This analysis demonstrates the utility of social network analysis in identifying influential actors and enhancing stakeholder engagement in public health initiatives.

Table 3 provides an overview of the key structural metrics for the social network of stakeholders involved in brucellosis prevention programs in Lorestan province. These metrics summarize the network's overall properties and reveal insights into the connectivity, cohesion, and efficiency of stakeholder interactions. The network consists of 18 unique stakeholders (nodes), each representing a key actor in the brucellosis prevention program. These stakeholders are connected through 42 edges, signifying relationships or interactions relevant to the program’s objectives. The density of the network is 0.2745, indicating that approximately 27.45% of all possible connections between the stakeholders are present. This suggests a moderately connected network where not all stakeholders are directly linked to one another. The average clustering coefficient is 0.2839, reflecting the tendency of stakeholders to form tightly knit groups or clusters. This value suggests a moderate level of clustering within the network, indicating localized collaboration among subsets of stakeholders. On average, each stakeholder is connected to approximately 4.67 others, as shown by the average degree, highlighting the general level of stakeholder interaction within the network. Additionally, the network contains 14 triangles, representing sets of three stakeholders that are all mutually connected. These triangles highlight areas of strong collaboration or cohesion within the network. The diameter of the network is 3, meaning the longest distance between any two stakeholders is three steps or connections. This, combined with an average path length of 1.8954, shows that stakeholders are generally well-connected and can typically reach one another in fewer than two steps. Overall, these metrics illustrate the structure and dynamics of the stakeholder network, providing valuable insights into its potential effectiveness in facilitating collaboration, information flow, and coordinated efforts in brucellosis prevention.

**Table 3:** Key structural metrics of the stakeholder network

Metric	Value
Nodes	18
Edges	42
Density	0.2745
Average clustering coefficient	0.2839
Average degree	4.6667
Number of triangles	14
Diameter	3
Average path length	1.8954

**Fig. 5:**
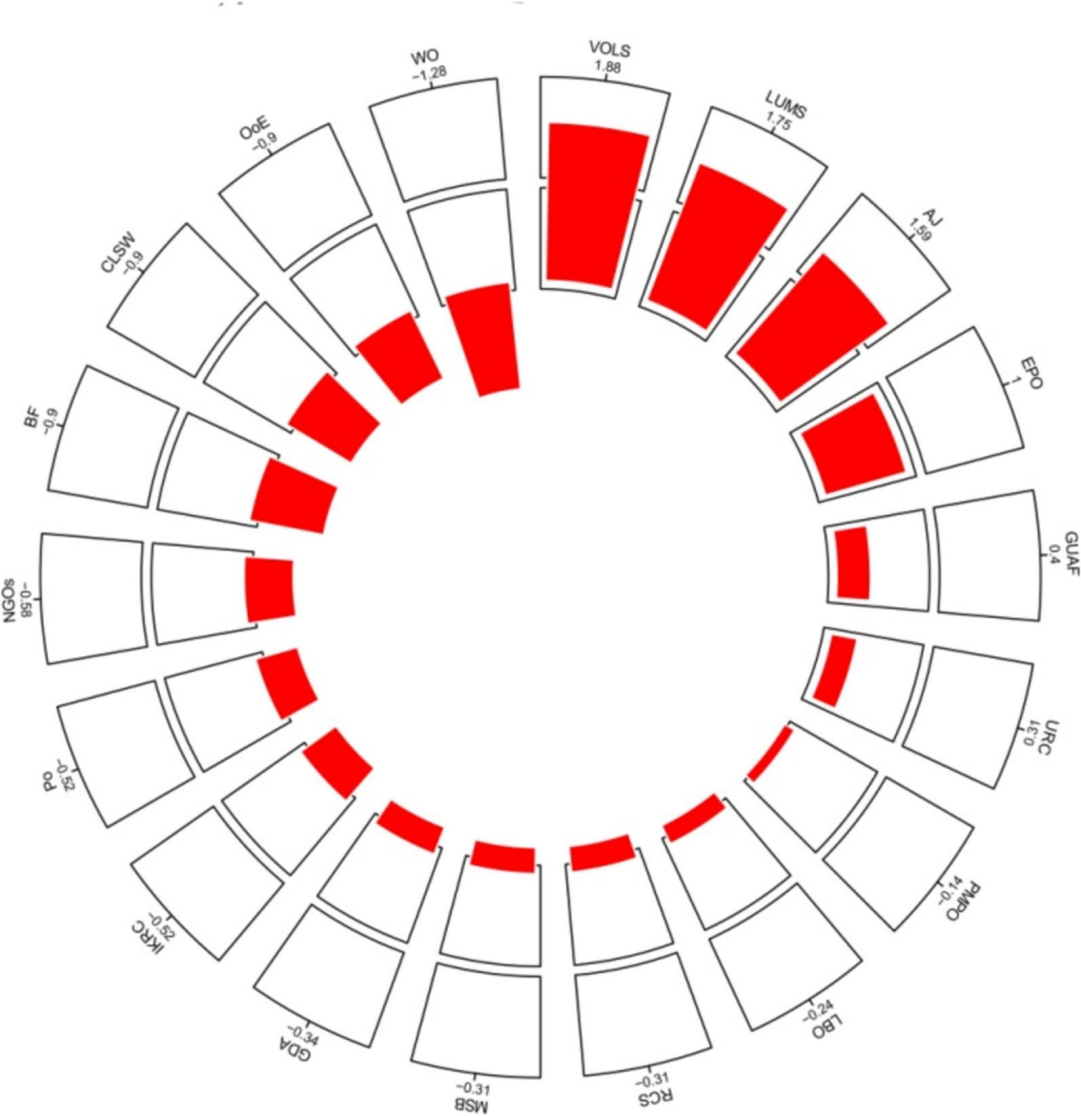
Most important actors based on composite score

Figure 5 highlights the most influential actors in the brucellosis prevention program in Lorestan Province, based on their composite scores. Composite scores were calculated by averaging the scores for influence, interest, power, and support, with equal weighting applied to each dimension.

Actors such as VOLS (1.88), LUMS (1.75), and AJ (1.39) exhibit the most prominent positive red bars, highlighting their roles as key contributors with strong positive influence in Brucellosis prevention efforts. Conversely, actors such as WO (-1.28), CLSW (-0.9), and MSS (-1.0) show negative values, suggesting they may negatively impact the outcome or hinder progress toward the goal. The apparent discrepancy between the SNA metrics (highlighting EPO and GUAF) and the composite scores (highlighting VOLS, LUMS, and AJ) can be explained by the fact that SNA metrics focus on network structure and connectivity, while composite scores reflect the overall influence, interest, power, and support of stakeholders. Both perspectives are complementary and provide a comprehensive understanding of stakeholder roles.

### Statistical validation and sensitivity analysis

We compared the observed network with 1000 randomized networks generated using the Erdős–Rényi model. The observed network density (0.2745) and clustering coefficient (0.2839) were significantly higher than those of the randomized networks (*p* < 0.001), indicating that the observed patterns of collaboration among stakeholders are non-random and reflect meaningful interactions. To evaluate the stability of the network, we systematically removed key stakeholders (e.g., VOLS, LUMS, EPO) and recalculated the network metrics. The results demonstrated that the removal of these stakeholders led to a measurable decrease in network density and centrality metrics, underscoring their critical roles in maintaining the network’s structure. However, the overall network properties remained stable, indicating that the findings are robust and not overly dependent on any single actor. These analyses confirm that the stakeholder network is both statistically significant and resilient to the removal of key players, reinforcing the reliability of our findings and their implications for brucellosis prevention efforts.

## Discussion

This study provides critical insights into the stakeholder network involved in brucellosis prevention in Lorestan province, Western Iran. The systemic, economic, and cultural challenges highlighted in this study were derived from the stakeholder scores and metrics, as well as contextual information provided by participants during the questionnaire and expert consultations. By combining these findings with broader contextual information, we can better understand the barriers to brucellosis prevention and identify actionable strategies for addressing this persistent public health issue. Below, we discuss the implications of our findings, compare them with successful models from other health systems, and propose recommendations for a National Brucellosis Control Program.

The SNA revealed that organizations such as the Veterinary Organization of Lorestan Province (VOLS), Lorestan University of Medical Sciences (LUMS), and the Ministry of Agriculture, Jahad (AJ) play central roles in brucellosis prevention efforts. These stakeholders exhibit high levels of influence, support, and interest, making them pivotal in shaping policies and implementing prevention programs. However, the limited involvement of NGOs and community-based organizations, such as the Barakat Foundation, underscores a critical gap in grassroots engagement. This finding aligns with broader challenges in Iran's health system, where top-down approaches often fail to incorporate local perspectives and resources [23].

The moderate network density (0.2745) and clustering coefficient (0.2839) observed in this study reflect fragmented coordination among stakeholders, exacerbated by the centralized structure of Iran’s health system. While organizations like VOLS and LUMS demonstrate strong commitment, the lack of intersectoral collaboration and resource allocation inefficiencies hinder their effectiveness. This fragmentation is particularly problematic in rural areas, where brucellosis remains endemic due to traditional livestock practices, limited access to veterinary services, and the consumption of unpasteurized dairy products [24]. The absence of a unified, multidisciplinary approach to disease control is a significant barrier, as evidenced by the limited involvement of NGOs and environmental agencies in the stakeholder network [5].

The persistence of brucellosis in Iran is deeply rooted in economic and systemic challenges. As highlighted in the provided context, economic sanctions and financial constraints have severely impacted the country’s ability to control the disease [25]. The high cost of importing livestock vaccines and the lack of domestic production have hindered vaccination efforts, while economic hardships have driven communities to rely on cheaper, unpasteurized dairy products. These factors, combined with the fragmented nature of Iran's health system, have created a perfect storm for the re-emergence and spread of brucellosis [26].

The centralized structure of Iran's health system further exacerbates these challenges [24]. While key organizations like VOLS and LUMS are committed to brucellosis prevention, the lack of intersectoral collaboration and resource allocation inefficiencies hinder their effectiveness. The absence of a unified, multidisciplinary approach to disease control is a significant barrier, as evidenced by the limited involvement of NGOs and environmental agencies in the stakeholder network [18].

Comparisons with health systems in other countries provide valuable lessons for Iran. Countries like Australia, the Netherlands, and the United States have successfully controlled brucellosis through robust surveillance systems, strict regulatory measures, and strong intersectoral collaboration [27–29]. Similarly, community-based interventions in low- and middle-income countries (LMICs) such as India and Kenya demonstrate the importance of participatory approaches and local engagement [30, 31]. India’s National Animal Disease Control Program (NADCP) leverages local networks and community health workers to improve vaccination coverage and public awareness, while Kenya’s One Health approach fosters collaboration between human health, animal health, and environmental sectors [30, 32]. These examples highlight the potential benefits of adopting similar strategies in Iran, particularly in high-risk regions like Lorestan province.

The One Health approach, which emphasizes the interconnectedness of human, animal, and environmental health, offers a promising framework for addressing brucellosis in Iran [33]. This study aligns with the principles of One Health by highlighting the need for greater collaboration among diverse stakeholders, including government agencies, veterinary services, environmental organizations, and community groups [34]. By adopting a One Health approach, Iran can address systemic barriers such as fragmented coordination, limited resource allocation, and insufficient public awareness [29].

For instance, integrating veterinary and public health services could improve disease surveillance and response times, while community engagement initiatives could enhance public awareness and compliance with prevention measures [35]. While this study did not involve WHO and FAO in the SNA, their endorsement of the One Health approach as a key strategy for combating zoonotic diseases like brucellosis underscores the importance of international collaboration. Implementing this approach in Iran would require policy reforms, capacity building, and sustained investment in intersectoral collaboration [36].

### Recommendations for a National Brucellosis Control Program

Based on the findings of this study and the broader context of Iran’s health system, we propose the following recommendations for a National Brucellosis Control Program:

- Adopt a One Health framework: Establish a formalized One Health framework to guide collaboration among human health, animal health, and environmental sectors. This framework should include integrated surveillance systems, shared databases, and real-time information exchange to enhance early detection and response to brucellosis outbreaks.
- Strengthen intersectoral collaboration: Create a multisectoral task force for brucellosis prevention, involving key stakeholders such as the Ministry of Health, Ministry of Agriculture, Veterinary Organization, environmental agencies, and NGOs. This task force should develop and implement coordinated strategies for disease control.
- Enhance community engagement: Increase the involvement of community-based organizations, local leaders, and NGOs in brucellosis prevention efforts. This could include training community health workers, leveraging local networks for public awareness campaigns, and promoting safe animal-handling practices.
- Improve resource allocation: Prioritize funding for brucellosis prevention programs in high-risk areas, with a focus on improving access to veterinary services, diagnostic tools, and vaccination programs. Address economic barriers by subsidizing the cost of vaccines and pasteurized dairy products.
- Leverage international support: Collaborate with international organizations such as WHO and FAO to access technical expertise, funding, and best practices for brucellosis control. This could include partnerships for vaccine procurement, capacity building, and data management.
- Address economic and cultural barriers: Implement targeted interventions to address the economic and cultural factors that perpetuate brucellosis transmission. This could include public awareness campaigns to promote the consumption of pasteurized dairy products and subsidies to make these products more affordable.

### Limitations

While this study provides valuable insights into the stakeholder network involved in brucellosis prevention in Lorestan province, several limitations should be acknowledged. First, the study relied on self-reported data from a purposive sample of 58 experts (out of 75 invited), which may introduce selection bias and limit the generalizability of the findings. Second, the geographic focus on Lorestan province may not reflect the stakeholder dynamics in other regions of Iran. Third, the cross-sectional design of the study limits the ability to assess changes in stakeholder relationships over time. Future research should address these limitations by expanding the geographic scope, incorporating longitudinal data, and integrating qualitative methods to capture the nuances of stakeholder interactions.

## Conclusion

The control of brucellosis in Iran requires a comprehensive, multisectoral approach that addresses the systemic, economic, and cultural barriers to disease prevention. By adopting a National Brucellosis Control Program based on the One Health framework, Iran can strengthen intersectoral collaboration, enhance community engagement, and improve resource allocation. The findings of this study, combined with lessons from successful disease control programs in other countries, provide a roadmap for addressing the persistent challenge of brucellosis in Iran. Such efforts will not only reduce the burden of brucellosis, but also contribute to the broader goal of improving public health outcomes in high-risk regions like Lorestan province.

## Supplementary Information

Additional file1: Scores for influence, interest, level of support and power stakeholders in Brucellosis prevention programs in Lorestan province, Western Iran

## Data Availability

The datasets generated and/or analysed during the current study are not publicly available due to privacy or ethical restrictions but are available from the corresponding author on reasonable request.

## Abbreviations

SNA: Social network analysis
MoHME: Ministry of Health and Medical Education
WHO: World Health Organization
FAO: Food and Agriculture Organization
SID: Scientific Information Database
NGOs: Non-governmental organizations
LMICs: Low- and middle-income countries
NADCP: National Animal Disease Control Program
